# Long-term survival of LGR5 expressing supporting cells after severe ototoxic trauma in the adult mouse cochlea

**DOI:** 10.3389/fncel.2023.1236894

**Published:** 2023-08-24

**Authors:** Natalia Smith-Cortinez, Ferry G. J. Hendriksen, Dyan Ramekers, Robert J. Stokroos, Huib Versnel, Louise V. Straatman

**Affiliations:** ^1^Department of Otorhinolaryngology-Head and Neck Surgery, University Medical Center Utrecht, Utrecht, Netherlands; ^2^UMC Utrecht Brain Center, University Medical Center Utrecht, Utrecht University, Utrecht, Netherlands

**Keywords:** inner ear regeneration, hearing loss, LGR5+ supporting cells, ototoxicity, adult mammalian cochlea

## Abstract

**Introduction:**

The leucine-rich repeat-containing G-protein coupled receptor 5 (LGR5) is a tissue resident stem cell marker, which it is expressed in supporting cells (SCs) in the organ of Corti in the mammalian inner ear. These LGR5+ SCs can be used as an endogenous source of progenitor cells for regeneration of hair cells (HCs) to treat hearing loss and deafness. We have recently reported that LGR5+ SCs survive 1 week after ototoxic trauma. Here, we evaluated Lgr5 expression in the adult cochlea and long-term survival of LGR5+ SCs following severe hearing loss.

**Methods:**

Lgr5GFP transgenic mice and wild type mice aged postnatal day 30 (P30) and P200 were used. P30 animals were deafened with a single dose of furosemide and kanamycin. Seven and 28 days after deafening, auditory brainstem responses (ABRs) were recorded. Cochleas were harvested to characterize mature HCs and LGR5+ SCs by immunofluorescence microscopy and quantitative reverse transcription PCR (q-RT-PCR).

**Results:**

There were no significant age-related changes in Lgr5 expression when comparing normal-hearing (NH) mice aged P200 with P30. Seven and 28 days after ototoxic trauma, there was severe outer HC loss and LGR5 was expressed in the third row of Deiters’ cells and in inner pillar cells. Seven days after induction of ototoxic trauma there was an up-regulation of the mRNA expression of Lgr5 compared to the NH condition; 28 days after ototoxic trauma Lgr5 expression was similar to NH levels.

**Discussion:**

The presence of LGR5+ SCs in the adult mouse cochlea, which persists after severe HC loss, suggests potential regenerative capacity of endogenous cochlear progenitor cells in adulthood. To our knowledge, this is the first study showing not only long-term survival of LGR5+ SCs in the normal and ototoxically damaged cochlea, but also increased Lgr5 expression in the adult mouse cochlea after deafening, suggesting long-term availability of potential target cells for future regenerative therapies.

## 1. Introduction

Inner ear regeneration has been extensively studied and described in non-mammalian species like birds and fish (Brignull et al., 2009). Notably, 1 week after damaging auditory epithelium from adult chickens, hair cells (HCs) start to regenerate spontaneously and within 2 months hearing function is completely restored (Smolders, 1999). In contrast, mammalian species do not spontaneously regenerate lost HCs. However, it has been recently described that supporting cells (SCs) in the cochlea of neonatal and adult mice act as endogenous otic progenitor cells and express the stem cell markers leucine-rich repeat-containing G-protein coupled receptor 5 (LGR5) (Groves, 2010; Shi et al., 2012, 2013; Bramhall et al., 2014; Zak et al., 2016; Smith-Cortinez et al., 2023), LGR4 (Zak et al., 2016), and SOX2 (Hume et al., 2007; Dabdoub et al., 2008).

In the embryonic mouse cochlea, LGR5 is expressed in the whole organ of Corti, in HCs and SCs. Later in development LGR5 expression gradually decreases, and at postnatal day 1 (P1) LGR5 is expressed in the greater epithelial ridge (GER), third row of Deiters’ cells (DC3s), inner border cells and inner pillar cells (IPCs) (Chai et al., 2011; Shi et al., 2012). Afterward, expression further decreases and at P30 LGR5 is restricted to DC3s and IPCs (Chai et al., 2011; Shi et al., 2012; Smith-Cortinez et al., 2021). It has only recently been described, also by us, that LGR5+ SCs are present in the cochlea of adult mice up to postnatal day 100 (P100) in DC3s and IPCs (Shi et al., 2012; Smith-Cortinez et al., 2021). LGR4 and SOX2 are expressed in all DC3s in the adult mice (Oesterle et al., 2008; Oesterle and Campbell, 2009; Zak et al., 2016).

Leucine-rich repeat-containing G-protein coupled receptor 5, LGR4, and SOX2 positive SCs in the cochlea are known to give rise to HCs during embryonic development (Jacques et al., 2012; Shi et al., 2012; Zak et al., 2015, 2016). This process is controlled by the Wnt, Notch, Sonic hedgehog (Shh), fibroblast growth factor (FGF), and bone morphogenetic proteins (BMP)/transforming growth factor-β (TGF-β) signaling pathways by upregulating *Atoh1*, a key transcription factor in HC differentiation (Li et al., 2005; Pujades et al., 2006; Driver et al., 2008; Zak et al., 2015; Ebeid and Huh, 2017; Ellis et al., 2019; Smith-Cortinez et al., 2023). These signaling pathways have also been described to participate in non-mammalian inner ear spontaneous regeneration (Brignull et al., 2009; Jacques et al., 2014; Jiang et al., 2014) and are the focus of interest for promoting cochlear regeneration in mammalian species (Smith-Cortinez et al., 2023). In order to promote HC regeneration in mammals as a treatment for hearing impairment, it is important to establish whether target cells for future regenerative therapies (i.e., SCs with potential progenitor capacity) survive after trauma.

It is known that SOX2 positive SCs are present in adult mice and survive up to a year after severe ototoxic trauma (Oesterle et al., 2008; Oesterle and Campbell, 2009). We have previously shown that LGR5+ SCs are present in the cochlea of adult mice and that they survive 7 days after ototoxically induced HC loss (Smith-Cortinez et al., 2021). In order to translate these findings to patients that have had hearing loss for a long time, evaluating long-term survival of LGR5+ SCs after trauma is crucial. Therefore, in this study, we investigated the survival of LGR5-expressing SCs in mice 28 days after induction of severe hearing loss with kanamycin and furosemide. In addition, we determined the expression levels of HC-specific genes: *Myo7A*, *Slc26a5*, and *Otof* and the progenitor cell-specific genes Lgr5, Lgr4, and Sox2 in the mouse cochlea 7 and 28 days after the induction of severe hearing loss.

## 2. Material and methods

### 2.1. Animals

We used 33 P30 heterozygous LGR5-EGFP-CreERT2 (The Jackson Laboratory, Bar Harbor, ME, USA, Stock 008875) mice (Lgr5GFP, 13 females and 20 males), 5 P200 Lgr5GFP mice (3 females and 2 males) and 6 P200 C57BL/6 wild type (WT) mice (all females). Mice were housed in open cages with food and water *ad libitum* and standard laboratory conditions. All surgical and experimental procedures were approved by the Dutch Central Authority for Scientific Procedures on Animals (CCD:1150020186105). In contrast to our previously described protocol (Smith-Cortinez et al., 2021), we used both male and female mice randomized for experiments. This avoided the scarification of female mice without being used for experiments and therefore reduce the total number of experimental animals used.

### 2.2. Genotyping

Lgr5GFP transgenic mice were genotyped by isolating DNA from ear tissue as previously described (Smith-Cortinez et al., 2021). Genomic DNA isolation was performed with DirectPCR lysis reagent (Viagen, Biotech, Los Angeles, CA, USA, 402-E) according to the manufacturer’s instructions. The primers for PCR amplification were: GFP, forward: CACTGCATTCTAGTTGTGG; and reverse: CGGTGCCCGCAGCGAG. Amplicons were separated by electrophoresis in a 3% agarose gel.

### 2.3. Deafening procedure

Non-treated mice were used as normal-hearing (NH) controls: 4 Lgr5GFP P30 mice for histology (2 females and 2 males), 8 Lgr5GFP P30 mice for RNA isolation (4 females and 4 males), 5 Lgr5GFP P200 mice for RNA isolation (3 females and 2 males) and 6 P200 WT mice for RNA isolation (all females). Lgr5GFP mice were deafened by kanamycin sulfate (Sigma-Aldrich, St. Louis, MO, USA, 60615) and furosemide (Centrafarm, Etten-Leur, Netherlands). A subcutaneous injection of kanamycin sulfate (700 mg/kg to male mice and 900 mg/kg to female mice, stock solution 100 mg/ml in saline) was followed by a tail vein injection of 100 mg/kg furosemide (stock solution 100 mg/ml) performed under anesthesia 5 min after kanamycin injection to induce ototoxicity. Different concentrations of kanamycin were used for male and female mice because we have observed 700 mg/kg kanamycin which induces HC loss in most male mice (Jansen et al., 2013; Smith-Cortinez et al., 2021) does not induce HC loss to the same extent in female mice (unpublished data). Deafened mice were examined 7 and 28 days after injections: for histology 4 mice at 7 days (all males) and 4 mice at 28 days (2 females and 2 males); for RNA isolation 5 mice at 7 days (1 female and 4 males) and 8 mice at 28 days (2 females and 6 males). Mice were weighed before the deafening procedure and weekly after the deafening, as part of welfare monitoring.

### 2.4. Auditory brainstem responses

Auditory brainstem responses (ABRs) were recorded under isoflurane anesthesia in a soundproof and electrically shielded box (52 × 34 × 28 cm). Horizon™ multiLead™ ribbon subdermal needle electrodes (3) 27 ga needle, 13 mm (Rochester Horizon) (3) were placed behind the right pinna (active), on the skull (reference), and in the hind limb (ground) using a subdermal approach. Acoustic stimuli consisting of 20-μs monophasic clicks were generated and attenuated using a TDT3 system (Multi-I/O processor RZ6; Tucker-Davis Technologies, Alachua, FL, USA), and presented in free field using a Bowers & Wilkins speaker (CCM683; 8 Ω; 25–130 W) at 5 cm distance from the right ear. The electrode signals were pre-amplified using a Princeton Applied Research (Oak Ridge, TN, USA) 5113 pre-amplifier (amplification × 5,000; band pass filter 0.1–10 kHz). The amplified signal was digitized by the same TDT3 system for analysis (100 kHz sampling rate, 24-bit sigma-delta converter). The responses were averaged over 500 repetitions (maximum) and stored on a PC for offline analysis with custom MATLAB software. The sound level was attenuated in 10 dB steps, starting with maximum sound level at approximately 105 dB peak equivalent SPL, until 10 dB below the sound level with no visible ABR response. The threshold was defined as the interpolated sound level at which the amplitude of the largest ABR wave was 0.3 μV. ABRs were recorded before deafening, and 7 and 28 days after deafening. Mice with significant residual hearing (threshold shifts <25 dB) were excluded from the analyses.

### 2.5. Cryosectioning and whole mount sample preparation

Mouse cochleas were harvested after termination by decapitation and processed for cryosectioning and whole-mount preparations as described previously (Smith-Cortinez et al., 2021). Briefly, after fixation (2% paraformaldehyde, Sigma-Aldrich, 158127) cochleas were decalcified at room temperature for 7 days. Cryopreservation was performed by a sucrose gradient after which tissues were embedded in OCT compound (Sakura Finetek Europe B.V., Alphen aan den Rijn, Netherlands, 6200) and stored at −80°C. Cryosections of 12 μm were cut using a Leica CM3050 cryostat and mounted on microscope slides. For whole-mount samples, cochleas were fixed and the otic capsule was opened, the lateral wall, Reissner’s membrane, tectorial membrane and modiolus were removed and the basilar membrane carrying the organ of Corti was dissected into individual half-turns.

### 2.6. Immunofluorescence microscopy

Immunofluorescence staining was performed on cryosections and whole-mount dissections. The tissues and slides were washed with blocking solution [2% donkey serum (Sigma-Aldrich, S30), 5% fetal bovine serum (FBS, Sigma-Aldrich, F9665), and 0.1–0.5% triton X-100 (Sigma-Aldrich, X100) in PBS]. Specimens were incubated with primary antibodies, anti-myosin VIIA (MYO7A, 1/200, rabbit, Proteus Biosciences, 25-6790) and anti-GFP (1/200, goat, Abcam, ab5450) overnight at 4°C. Later, slides and tissues were washed with blocking solution and incubated with secondary antibodies donkey-anti Rabbit-Alexa 594 (1/500, Invitrogen, A-21207), donkey-anti Goat-Alexa 488 (1/500, Abcam, AB150129), and DAPI solution (1/500, Abcam, AB228549) for 90 min at room temperature. Lastly, specimens were washed in PBS and mounted in Vectashield Antifade Mounting Medium (Vector laboratories, H-1000). Slides were imaged using a Zeiss LSM700 Scanning Confocal Microscope. Three-dimensional image reconstructions of *Z*-stacks were performed using ImageJ software.

### 2.7. RNA isolation, cDNA synthesis, and real-time quantitative PCR

RNA isolation of cochlear tissue was performed as previously described (Vikhe Patil et al., 2015). Briefly, 2 cochleas per mice were pooled to get enough RNA to analyze gene expression. Cochleas were snap frozen in liquid nitrogen upon collection and RNA was isolated when all mice had been sacrificed. Cochleas were triturated in TRIzol (Thermo Fisher Scientific, Amsterdam, Netherlands, 15596026) and a chloroform extraction was performed. To purify the RNA a column-based extraction protocol was performed (Direct-Zol RNA mini prep, ZymoResearch, R2050). Quality and quantity of RNA were determined using a NanoDrop 2000c UV-vis spectrophotometer (Thermo Fisher Scientific). cDNA synthesis was performed from 2.5 μg RNA using random nonamers (Sigma-Aldrich, R7647) and 200 U/μl of M-MLV reverse transcriptase (Invitrogen, Waltham, MA, USA, 28025013) in 50 μl reactions. TaqMan gene expression assays were purchased in Thermo Fisher Scientific for Myo7A (Mm01274015_m1), Slc26a5 (Mm00446145_m1), Otof (Mm00453306_m1), Lgr5 (Mm00438890_m1), Lgr4 (Mm00554385_m1), Sox2 (Mm03053810_s1), and 18S (Mm04277571_s1). All target genes were amplified using the TaqMan™ Fast Advanced Master Mix (Thermo Fisher Scientific, 4444556) on a Bio-Rad MyI (Bio-Rad). Expression of genes is presented in 2^–ΔΔCT^ or fold induction and normalized to 18S.

### 2.8. Statistical analysis

Differences in mRNA expression in cochlear tissues between the deafened and NH mice were evaluated by performing a one-way ANOVA followed by a Tukey’s multiple comparisons test. These analyses were performed in GraphPad Prism version 8.0.0 for Windows (GraphPad Software, San Diego, CA, USA). Results were considered statistically different when *p* < 0.05.

## 3. Results

### 3.1. Lgr5 is expressed in mature adult normal-hearing WT and Lgr5GFP transgenic mice

**FIGURE 1:**
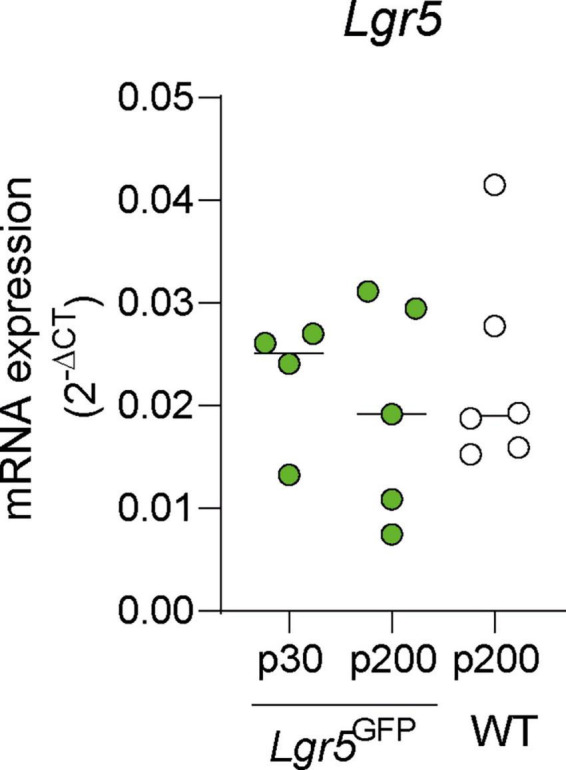
mRNA expression in mature adult normal-hearing Lgr5GFP and WT mice. Quantification of mRNA expression of *Lgr5* (versus *18S*) in P30 Lgr5GFP transgenic mice, P200 Lgr5GFP transgenic mice, and P200 WT mice. nLgr5-GFP(P30) = 5; nLgr5GFP(P200) = 5 nWT(P200) = 6.

We evaluated the mRNA expression of Lgr5 in Lgr5GFP NH mice aged P30 and P200, compared to NH WT mice aged P200. We observed no significant differences in the mRNA expression of Lgr5 in the cochlea of P30 Lgr5GFP, P200 Lgr5GFP, or P200 WT mice (Figure 1, *p* = 0.94 Kruskal–Wallis test). These results show preservation of LGR5+ SCs in adulthood.

### 3.2. LGR5-positive supporting cells survive 28 days after deafening in the mouse cochlea

**FIGURE 2:**
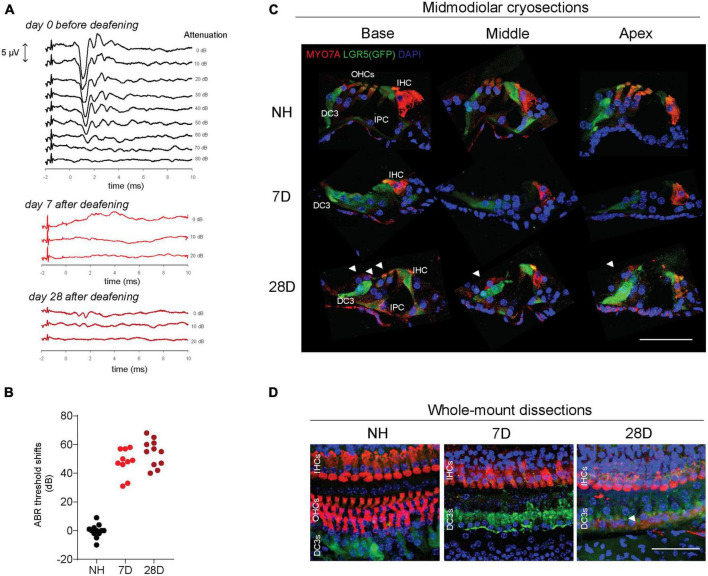
LGR5+ supporting cells are present long-term after ototoxic treatment. **(A)** Representative auditory brain stem responses (ABRs) of one Lgr5GFP mouse before deafening (top), and 7 days (7D, middle) and 28 days (28D) after deafening (bottom). ABRs were performed to click stimuli with 10 dB attenuation steps. **(B)** ABR threshold shifts from normal-hearing (NH) Lgr5GFP mice, and Lgr5GFP mice 7 days after ototoxicity (7D) and 28 days after ototoxicity (28D) nNH = 12, n7D = 10, n28D = 11. **(C)** Representative images of immunofluorescence microscopy of midmodiolar cryosections of the cochlea of NH, D7, and D28 Lgr5GFP mice stained with myosin VII A (MYO7A) in red, GFP (LGR5) in green and DAPI in blue. **(D)** Representative images of immunofluorescence microscopy of cochlear whole-mount dissections of NH, 7D, and 28D Lgr5GFP mice stained with MYO7A in red, GFP (LGR5) in green and DAPI in blue. Bar, 50 μm. nNH = 3, n7D = 4, n28D = 4 (representative images of 1 cochlea per group). IHC, inner hair cell; OHC, outer hair cells; DC3, third row of Deiters’ cells; IPC, inner pillar cell.

All Lgr5GFP transgenic mice had NH thresholds (on average 37 dB peak equivalent sound pressure level, peSPL) as observed in click-evoked ABRs (Figures 2A, B). As intended, 7 and 28 days after ototoxic trauma mice only showed click-evoked ABRs at high levels (Figure 2A). Seven and 28 days after ototoxic trauma large ABR threshold shifts were shown (Figure 2B, 7D: 48 ± 9 dB, and 28D: 54 ± 9 dB), while the NH control animals did not show any threshold shifts (0 ± 4 dB).

Immunofluorescence microscopy of midmodiolar cryosections showed that in the cochlea of NH mice, myosin VII A (MYO7A) expression was observed in inner hair cells (IHCs) and outer hair cells (OHCs), as expected, and LGR5 (GFP) expression was restricted to DC3 and IPCs in the base, middle and apex (Figure 2C, top row). One week after deafening MYO7A expression was observed throughout the cochlea in IHCs only. In the area of the OHCs MYO7A staining was not visible; nuclei (observed by DAPI staining) were also not visible, confirming all OHCs died due to the ototoxic treatment. LGR5 (GFP) expression was mostly observed in DC3 and, to a lesser extent in IPCs 1 week after deafening (Figure 2C, middle row). Interestingly, 28 days after deafening MYO7A expression was observed not only in IHCs and but also in OHCs (white arrowheads, Figure 2C, bottom row). These cells were found in all (4) mice evaluated in this group and were located in the outer side of LGR5+ DC3s (toward the stria vascularis) and at a larger distance from the IHC, compared to the original position of the OHCs. LGR5 (GFP) expression was still detected in DC3s and IPCs (Figure 2C, bottom row). Interestingly, we observed LGR5+ cells in the lateral wall of the cochlea in the region of the spiral prominence in NH mice and in mice 7 and 28 days after deafening (Supplementary Figure 1). Immunofluorescence microscopy of cochlear whole-mounts (Figure 2D) confirmed the presence of MYO7A expressing IHCs and LGR5 expressing DC3s and IPCs in the cochlea of animals 7 and 28 days after deafening. It furthermore showed complete loss of OHCs 7 days after deafening, and it showed some MYO7A+ cells located near LGR5+ DC3 suggestive of OHCs 28 days after deafening (white arrowheads, Figure 2D). In summary, these results show long-term survival of LGR5+ SCs in IPCs and DC3 after severe ototoxic trauma.

### 3.3. *Lgr5* expression is enhanced 7 days after ototoxic trauma in the mouse cochlea

**FIGURE 3:**
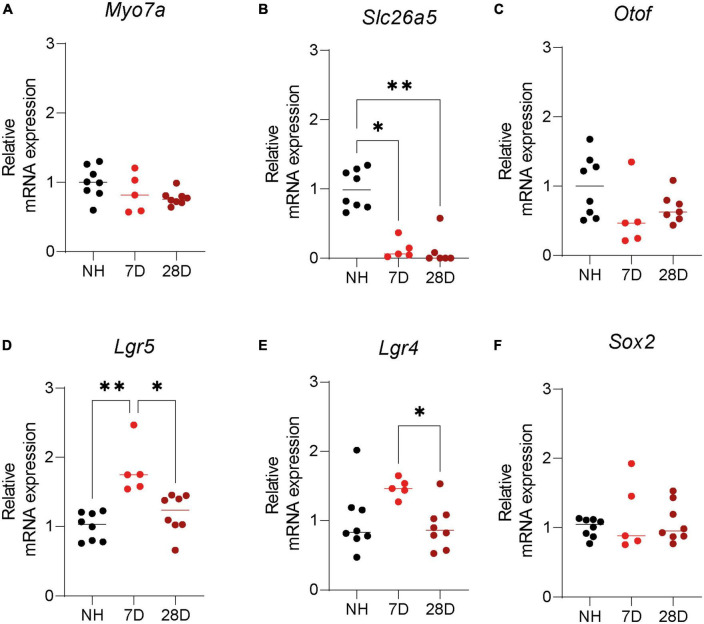
Expression of hair cell and supporting cell markers in the cochlea of normal hearing and deafened mice. Quantification of mRNA expression of **(A)** *Myo7A*, **(B)** *Slc26a5*, **(C)** *Otof*, **(D)** *Lgr5*, **(E)** *Lgr4*, and **(F)** *Sox2* in normal hearing (NH) Lgr5GFP transgenic mice and Lgr5GFP mice 7 days after ototoxicity (7D) and 28 days after ototoxicity (28D). nNH = 8, n7D = 5, n28D = 7.

Immunofluorescence microscopy analysis clearly showed survival of LGR5+ SCs in the cochlea of short- and long-term deafened mice. Still, quantification of LGR5 is difficult using imaging techniques due to many variables such as antibody binding and fluorophore quenching. For that reason, we evaluated expression of key HC and progenitor cell genes in the cochlea of NH mice and mice 7 and 28 days after induction of ototoxic trauma. Analysis of HC genes showed *Myo7a*, the gene encoding myosin VII A, was slightly (20–30%) but not significantly reduced in the cochlea of mice 7 and 28 days after deafening compared to NH mice (Figure 3A, *p* = 0.11 one-way ANOVA test). Prestin, which is encoded by *Slc26a5*, is expressed in OHCs and was significantly reduced (virtually vanished) in the cochlea of mice 7 and 28 days after deafening compared to NH mice (Figure 3B, *p* < 0.0001 one-way ANOVA test, 7D vs. NH: *p* < 0.0001; 28D vs. NH: *p* < 0.0001 Turkey’s multiple comparisons test). *Otof*, the gene encoding otoferlin, a protein expressed by IHCs, was reduced by about 50% but not significantly 7 or 28 days after ototoxic treatment compared to NH mice (Figure 3C, *p* = 0.12 one-way ANOVA test).

Surprisingly, *Lgr5* expression was significantly upregulated 7 days after ototoxic treatment (by more than 50%) and no differences were found 28 days after ototoxic treatment compared to NH mice (Figure 3D, *p* = 0.0002 one-way ANOVA test, 7D vs. NH: *p* = 0.0002; 28D vs. NH: *p* = 0.41; 7D vs. 28D: *p* = 0.0025 Turkey’s multiple comparisons test). Also the *Lgr4* expression was increased relative to the NH cochlea 7 days after deafening (but not significantly) and back to NH levels after 28 days (Figure 3E, *p* = 0.035 one-way ANOVA test, NH vs. 7D: *p* = 0.084; 28D vs. NH: *p* = 0.86 Turkey’s multiple comparisons test). *Lgr4* was significantly downregulated from 7 to 28 days (Figure 3E, 7D vs. 28D: *p* = 0.034 Turkey’s multiple comparisons test). *Sox2* expression was not changed 7 or 28 days after ototoxic treatment compared to NH mice (Figure 3F, *p* = 0.65 one-way ANOVA test). These results suggest ototoxic treatment promotes a specific increase in *Lgr4* and *Lgr5* mRNA expression 7 days after ototoxicity, which is back to NH levels long-term after deafening. In addition, it shows long-term survival of cells with progenitor capacity, expressing *Lgr5*, *Lgr4*, and *Sox2*, after deafening in the adult cochlea.

## 4. Discussion

Here, we evaluated expression of *Lgr5* and survival of LGR5+ SCs in cochleas of adult NH and deafened mice at various ages. The mRNA expression of Lgr5 was similarly expressed in cochleas of Lgr5GFP transgenic mice at ages P30 and P200, and moreover, it was similarly expressed in cochleas of Lgr5GFP transgenic and WT mice at age P200. Furthermore, to our knowledge we observed for the first time long term LGR5+ SC survival up to 28 days after ototoxic trauma in the cochleas of adult Lgr5GFP transgenic mice. Interestingly, the mRNA expression of *Lgr5* and, to a lesser extent, *Lgr4* but not *Sox2* was upregulated 7 days after deafening.

### 4.1. Endogenous otic progenitor cells in the adult mice

The presence of endogenous otic progenitors has been extensively evaluated in the neonatal mice. It is known SCs in neonatal mice express progenitor cell markers such as *Lgr5* and *Sox2* (Chai et al., 2011; Shi et al., 2012; Zak et al., 2016) and proliferate and transdifferentiate to HCs as part of the continued development of the inner ear. The expression of *Lgr5* and *Sox2* is maintained in the adult cochlea and has been recently reviewed by us (Smith-Cortinez et al., 2023). LGR5+ SCs are present in the adult cochlea up to P100 in DC3 and IPCs (Shi et al., 2012; Smith-Cortinez et al., 2021), and here we show for the first time that the mRNA expression of Lgr5 is maintained at least up to P200. We further demonstrate that the Lgr5 expression does not decrease as mice age, since the relative expression of Lgr5 was not downregulated in P200 compared to P30 mice. This suggests that Lgr5 expression might be maintained throughout adulthood. Interestingly, we have for the first time shown that there are LGR5+ cells in the lateral wall in the area of the spiral prominence in adult mice. These cells resemble root cells because they have similar cellular projections as root cells and it has been recently shown by single cell transcriptomics that root cells express *Lgr5* and *Rspo2* (Gu et al., 2020). Unfortunately, the role of LGR5 in these cells has not been established yet. It has also been described that SOX2, which is involved in neurogenesis and inner ear development (Kiernan et al., 2005; Hume et al., 2007), is present in the nuclei of all SC subtypes in the normal adult (up to at least 1 year old) mouse organ of Corti (Oesterle and Campbell, 2009). As expected and in accordance to previous studies we also found SOX2 expression in the adult mouse cochlea. Although the expression of progenitor cell markers has been described in the adult mice, the regenerative capacity of these cells has been scarcely demonstrated. Progenitor cells from the adult cochlea have been described to produce HCs *in vitro* (McLean et al., 2017; Senn et al., 2020) and *in vivo* (Mizutari et al., 2013; Deng and Hu, 2022), providing the first evidence for HC regeneration in adulthood; still, further studies are needed to demonstrate if LGR5+ or SOX2+ SCs have progenitor potential in adulthood.

### 4.2. IHC and OHC genes in the cochlea of deafened mouse

The evaluation of mRNA expression of key HC genes showed that prestin, encoded by *Slc26a5*, was substantially downregulated at 7 and 28 days after deafening. This is consistent with previous observations that in our hearing loss model OHCs are completely abolished by the ototoxic treatment (Jansen et al., 2013; Smith-Cortinez et al., 2021). Also, in accordance to our previous data we found survival of MYO7A+ IHCs (Smith-Cortinez et al., 2021). Interestingly, we observed some MYO7A+ cells near the LGR5+ DC3s only in the cochlea of mice 28 days after deafening, which could correspond to either surviving OHCs or to newly produced OHCs. The first option is not likely since we did not observe these cells 7 days after deafening and they appear to be present in a different location as the original OHCs. OHCs locate between IHCs and DC3s, and the MYO7A+ cells in the 28 day group locate near DC3s but to the stria vascularis. In mice, no evidence points in the direction of spontaneous HC regeneration after ototoxicity, suggesting the second hypothesis, newly formed OHCs, is likely not true. More analysis are needed to investigate if these cells also expresses prestin, a marker of OHCs.

Due to the survival of most IHCs, the mRNA expression of *Otof*, encoding for Otoferlin, was slightly but not significantly downregulated. In line, mRNA expression of *Myo7a*, the gene responsible for myosin VIIA production, was not reduced by the ototoxic treatment although most OHCs had died already 7 days after ototoxicity. This result might suggest that IHCs express more Myo7a than OHCs or that other *Myo7a* expressing cells are present in the cochlea of these mice. Unfortunately, there is limited data on the transcriptomic profiles of deafened mice compared to NH mice. For example, Bai et al. (2019) showed transcriptomic analysis of mouse cochleas suffering from gentamicin damage; in these animals most HCs in the base and middle parts were injured and lost and they showed only 40% reduction in mRNA expression of *Myo7a* compared to non-treated mice. The results in this paper and in ours suggest that *Myo7a* expression in ototoxic models is not substantially downregulated due to a large survival of IHCs.

### 4.3. Survival of LGR5+ SCs in the cochlea of deafened mice

Interestingly, we observed survival of LGR5+ SCs 28 days after ototoxic trauma, which was accompanied by severe loss of OHCs and corresponding hearing loss of about 50 dB. We have previously shown that LGR5+ SCs survive 7 days after severe ototoxic trauma (Smith-Cortinez et al., 2021) and others have shown that SOX2+ SCs survive 1 year after ototoxic trauma even when no IHCs were present and a flat epithelium was observed (Oesterle and Campbell, 2009). No other studies have evaluated the presence of SCs with progenitor cell markers after deafening in adult specimens. One week after ototoxicity, we observed enhanced *Lgr5* mRNA expression (and to lesser extent for Lgr4) but no changes in *Sox2* mRNA expression. This suggests that in the cochlea, LGR5+ cells respond after ototoxic damage by increasing mRNA expression of this gene. This could suggest an initial response to activate the Wnt signaling pathway and promote proliferation. It has been described that 16 genes in the Wnt signaling pathway were differentially expressed between non-treated and gentamicin-damaged cochleas (Bai et al., 2019). Our experiments were performed on whole cochlear extracts, so the newly described population of LGR5+ cells found in the lateral wall (which survive in mice 7 and 28 days after deafening) contribute to the mRNA expression we quantify. To circumvent this issue further studies should focus on evaluating by single cell or single nuclei RNA sequencing the transcriptomic profile of SCs after deafening. In studies using neonatal mice, LGR5 expressing SCs have been described to differentiate to HCs *in vitro* and *in vivo* after the manipulation of Notch, Wnt, FGF, and BMP signaling pathways (Li et al., 2003; White et al., 2006; McLean et al., 2017; Samarajeewa et al., 2018; Bai et al., 2019; Ellis et al., 2019; Shu et al., 2019) even after HC loss. These results suggest that, although no spontaneous regeneration is observed in adult mammals, the LGR5+ SCs responsible for HC regeneration survive after HC loss and could therefore be targeted to promote hearing restoration.

### 4.4. LGR5+ SCs and their potential in HC regeneration during adulthood

It has been described that epigenetic modifications in the murine cochlea limit regenerative potential after P6 in mice due to the presence of repressive complexes that prevent transcription factors to reach target genes required for SC to HC transdifferentiation (Mittal et al., 2020; Tao et al., 2021). This might explain the lower regenerative potential observed in adult compared to neonatal mice. Interestingly, by targeting DNA methylases, which enables SC to HC transdifferentiation by targeting the epigenetic barrier, after ototoxically induced hearing loss, there is HC regeneration with improved ABR and DPOAE thresholds in adult mice (Deng and Hu, 2022). These authors had previously demonstrated that HCs were *de novo* produced from SOX2+ SCs after 5-azacytidine treatment (Deng and Hu, 2020). These studies and the current study suggest not only that SCs with progenitor potential survive severe trauma, but also that these cells are able to proliferate and differentiate *in vivo* to promote HC regeneration and functional hearing recovery in adulthood.

Few studies have evaluated HC regeneration and hearing improvement in patients. In the first clinical trial on this topic, adult patients suffering from mild to moderate sensorineural hearing loss (SNHL) were treated with a combination of drugs that activate the Wnt signaling pathway and target the epigenetic barrier to allow SC to HC transdifferentiation. Speech recognition in quiet and in background noise was improved 90 days after treatment (McLean et al., 2021), suggesting that Wnt responsive progenitor cells are present in the cochlea of adults with SNHL. Although these results are promising, in animal models spontaneous functional recovery has been observed after ototoxic trauma (Klis et al., 2002; Versnel et al., 2007) and due to the large variability in patients hearing impairment (from mild to moderate) it is possible that recovery could have also occurred without treatment, especially since baseline data differed between placebo and treatment groups. On the same topic, an ongoing clinical trial (REGAIN^1^) has reported positive results from a phase I safety study of a novel Notch inhibitor (LY3056480) in patients with mild to moderate SNHL and will evaluate pure-tone hearing thresholds and speech-in-noise perception. The results of this study will be interesting to see, especially since no drugs are used to enable epigenetic regulation preventing HC regeneration in adults. It is more likely that combined strategies are necessary, targeting the endogenous progenitor cells by manipulation of several key signaling pathways and the epigenetic control (Smith-Cortinez et al., 2023).

## 5. Conclusion

Our study showed for the first time the expression of Lgr5 in mice up to P200, which confirms potential endogenous progenitor cells are present during adulthood. We further demonstrated for the first time the survival of LGR5+ SCs 28 days after severe HC loss. Moreover, we demonstrated the mRNA expression of Lgr5 is specifically enhanced 7 but not 28 days after onset of cochlear trauma, possibly giving a therapeutic window to treating hearing loss in the future. Overall, these results suggest target cells for future regenerative therapies, i.e., SCs with progenitor cell markers (Lgr5, Lgr4, and Sox2) are present in adulthood and survive severe trauma.

## Data availability statement

The raw data supporting the conclusions of this article will be made available by the authors, without undue reservation.

## Ethics statement

The animal study was approved by the Dutch Central Authority for Scientific Procedures on Animals. The study was conducted in accordance with the local legislation and institutional requirements.

## Author contributions

NS-C and LS: conceptualization and writing—original draft. NS-C, FH, and HV: methodology. NS-C, FH, DR, and LS: investigation. NS-C and HV: formal analysis. HV, RS, and LS: resources, project administration, and funding. HV and LS: writing—revision and editing, and project supervision. All authors contributed to the article and approved the submitted version.

## Footnotes

1
https://www.regainyourhearing.eu/

## Supplementary material

Supplementary Figure 1 LGR5+ cells in the spiral prominence. Representative images of immunofluorescence microscopy of midmodiolar cryosections of the apex and base of the cochlea of Lgr5GFP mice before deafening [normal hearing (NH), **left**], and 7 days (7D, **middle**) and 28 days (28D, **right**) after deafening. Samples were stained with myosin VII A (MYO7A) in red, GFP (LGR5) in green and DAPI in blue. Bar, 50 μm. nNH = 3, n7D = 4, n28D = 4 (representative images of 1 cochlea per group). IHC, inner hair cell; OHCs, outer hair cells; DC3, third row of Deiters’ cells.
