# Supplementary figures and images for: Long-term survival of LGR5 expressing supporting cells after severe ototoxic trauma in the adult mouse cochlea
Source: Front Cell Neurosci. 2023 Aug 24;17:1236894. doi: 10.3389/fncel.2023.1236894 (PMC10483136; doi:10.3389/fncel.2023.1236894)

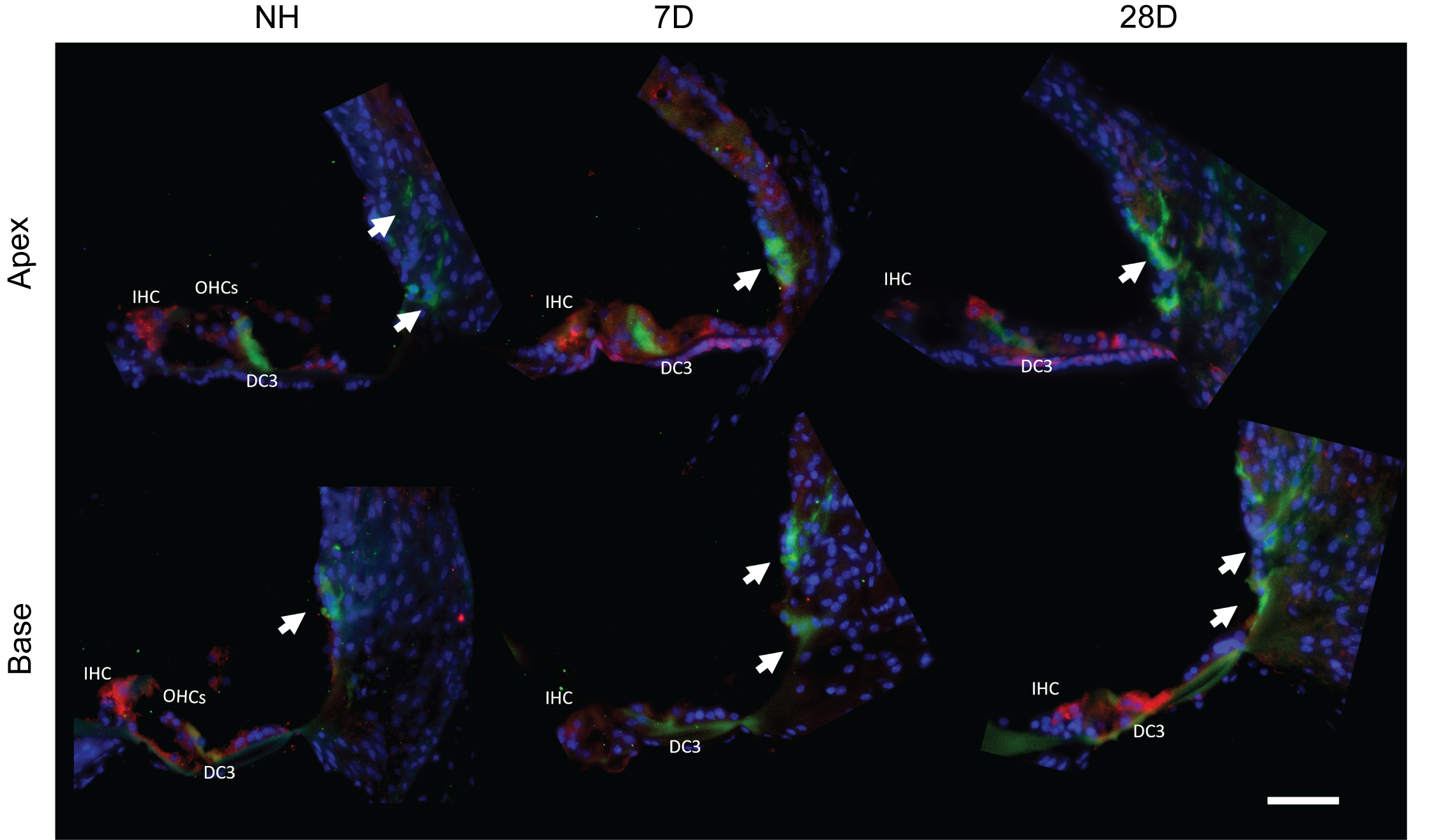

Supplement: Supplementary Figure 1 — LGR5+ cells in the spiral prominence. Representative images of immunofluorescence microscopy of midmodiolar cryosections of the apex and base of the cochlea of Lgr5GFP mice before deafening [normal hearing (NH), left], and 7 days (7D, middle) and 28 days (28D, right) after deafening. Samples were stained with myosin VII A (MYO7A) in red, GFP (LGR5) in green and DAPI in blue. Bar, 50 μm. nNH = 3, n7D = 4, n28D = 4 (representative images of 1 cochlea per group). IHC, inner hair cell; OHCs, outer hair cells; DC3, third row of Deiters’ cells. [file Image_1.TIF]
